# Olfactory Evoked Potentials and Brain MRI Outcomes in Multiple Sclerosis Patients: A Cross-Sectional Study

**DOI:** 10.3390/jcm14010141

**Published:** 2024-12-29

**Authors:** Rosella Ciurleo, Simona De Salvo, Fabrizia Caminiti, Annalisa Militi, Lilla Bonanno

**Affiliations:** IRCCS Centro Neurolesi Bonino Pulejo, 98124 Messina, Italy; rossella.ciurleo@irccsme.it (R.C.); fabrizia.caminiti@irccsme.it (F.C.); annalisa.militi@irccsme.it (A.M.); lilla.bonanno@irccsme.it (L.B.)

**Keywords:** multiple sclerosis, Olfactory Event-Related Potentials, magnetic resonance imaging, olfactory dysfunction, brain lesion load

## Abstract

**Background:** Olfactory dysfunction (OD) is an underestimated symptom in multiple sclerosis (MS). Multiple factors may play a role in the OD reported by MS patients, such as ongoing inflammation in the central nervous system (CNS), damage to the olfactory bulbs due to demyelination, and the presence of plaques in brain areas associated with the olfactory system. Indeed, neuroimaging studies in MS have shown a clear association of the OD with the number and activity of MS-related plaques in frontal and temporal brain regions. However, these studies have used only psychophysical tests to evaluate the OD in MS patients. Olfactory Event-Related Potentials (OERPs) are a method to assess olfaction with the clear advantage of its objectivity in comparison with psychophysical tests. The aim of this study was to investigate the association between the parameters of OERP components (latency and amplitude) and the lesion load of the brain regions which are involved in olfaction in a cohort of relapsing-remitting (RR) MS patients. **Methods:** In this cross-sectional study, we enrolled 30 RRMS patients and 30 healthy controls. The parameters of OERP components and magnetic resonance imaging data (lesions in the CNS) were analyzed in RRMS patients. **Results:** The association found between the RRMS patient groups with and without OERPs and the number of lesions in the frontal area as well as the correlation between the lesion load in the temporal area and OERP parameters suggest how brain alterations may impact on olfactory performance in MS. In addition, the predictive value of the number of lesions in the frontal and parietal areas for P2 amplitude also highlights the potential for OERP measures to serve as markers for disease progression in MS. **Conclusions:** This approach to assess the olfaction in MS could improve our understanding of the disease’s neurological impact and contribute to the development of new targeted interventions to mitigate olfactory sensory deficits.

## 1. Introduction

Multiple sclerosis (MS) is a chronic autoimmune neurological disorder marked by damage to myelin and nerve tissue in various parts of the central nervous system (CNS) including the brainstem, cortex, cerebellum, and spinal cord, resulting in a broad range of symptoms, such problems with vision, motor control, cognitive performance, balance, and sensation. Olfaction impairment has also been reported in patients with MS and, although it is a underestimated symptom compared to the others, it is able to affect patients’ quality of life because it is closely linked to their physical, behavioral, and cognitive conditions [1]. Olfactory dysfunction (OD) is frequently an initial significant sign of neurodegenerative diseases, such as Parkinson’s disease, Alzheimer’s disease, Huntington’s disease, and motor neuron disease [2]. It has recently been reported that the overall prevalence of OD among patients with MS is 27.2%, being more common in Italy at 69.6% and less common in Austria at 0.00% [3]. The most reliable psychophysical tests for evaluating human olfaction in a clinical setting are the University of Pennsylvania Smell Identification Test, the Connecticut Chemosensory Clinical Research Center Test, and the Sniffin’ Sticks Test. Nevertheless, the psychophysical assessments of odor identification and discrimination necessitate strong cognitive abilities that may be affected even during the initial phases of MS. Therefore, the objective evaluation of olfaction is one of the most challenging tasks in evaluating sensory functions. Upon the introduction of the Olfactory Event-Related Potentials (OERPs), it was possible to test olfactory nerve function and to objectively study the olfactory system. OERPs originate from the consecutive activation of various brain regions starting from the olfactory bulb and tracts, and including the orbitofrontal and insular cortices, as well as the rostrum-medial areas of the temporal lobe [2]. The initial OERP components (N1 and P1) signify the external cortical activity associated with sensory input detection and primary sensory processing. Conversely, the subsequent OERP components like P2 represent internal cortical activity connected to secondary cognitive processing and selective attention. Latency and amplitude are the main parameters of OERP components. The latency of N1 and P2 components measures the time needed for processing odor stimuli at sensory and cognitive levels. The amplitude indicates the importance of the stimulus and its informational value. The presence of OERPs is a good sign of normosmia, while the absence of OERPs indicates anosmia. Changes in the latency and/or amplitude of N1 and P2 components may indicate a case of reduced sense of smell (hyposmia). Preliminarily, it has been demonstrated that evaluating OD in SM with OERPs could serve as an unbiased indicator of neuroinflammation or neurodegeneration and a predictive factor for disability advancement [4,5]. In MS, several diagnostic criteria have been proposed and include clinical and instrumental data. The McDonald criteria, first introduced in 2001 and updated in 2005, 2011, and 2017, are frequently utilized [6]. The fundamental idea behind these criteria involves showing the dissemination over time (DIT) and space (DIS) of brain lesions (plaques) using clinical and/or magnetic resonance imaging (MRI) data. To sum up, to conclusively diagnose MS, there must be lesions present in at least two separate areas of the CNS (DIS) or the occurrence of lesions at different moments (DIT). MRI studies have demonstrated a clear association of OD with the number and activity of MS-related plaques in inferior frontal and temporal lobes [7,8,9]. However, these studies investigated OD by using psychophysical testing only and did not investigate the relationship between the OERPs and the alterations of olfactory brain structures. Only Holinski and coll [10] found a relationship between the olfactory bulb volume, the olfactory brain lesion load, and an increase in chemosensory potential latencies in MS patients. Because research on OEPRs and their association with brain alteration in MS is relatively scarce, in this study, we investigated the association between the parameters of OERP components (latency and amplitude) and the lesion load of the brain regions which are involved in olfaction in a cohort of relapsing-remitting (RR) MS patients. The use of electrophysiological methods combined with imaging methods in the evaluation of OD may contribute to a better understanding of this underestimated symptom in MS.

## 2. Materials and Methods

### 2.1. Study Population

In this cross-sectional study, 30 patients (19 females and 11 males) with a diagnosis of RRMS according to the revised 2011 McDonald criteria [11] and 30 healthy controls matched for age, sex, and smoking habits, without neurological or psychiatric disorders (18 females and 12 males), were recruited at IRCCS Centro Neurolesi “Bonino-Pulejo” of Messina, Italy. The Table 1 shows a more detailed description of demographic and clinical characteristics of patients and controls. All participants were carefully interviewed about their medical history to rule out diseases of the nasal and paranasal cavities as well as other factors affecting their sense of smell. Otolaryngological examination, through upper airway rhinoscopy, confirmed the patency of nasal cavities and ruled out any anatomic abnormalities. Subjects who were taking medications that could impact their sense of smell or the recording of OERPs were not included in the study. This includes antispasmodics, antidepressants, hypnotic-sedatives, and steroids. Due to this factor, we did not include patients who had undergone steroid treatment for MS relapses in the 3 months leading up to enrollment. We also excluded RRMS patients for whom MRI was contraindicated. Additional reasons for exclusion were other neurological diseases, pregnancy, or lactation.

### 2.2. Study Procedures

**Figure 1:**
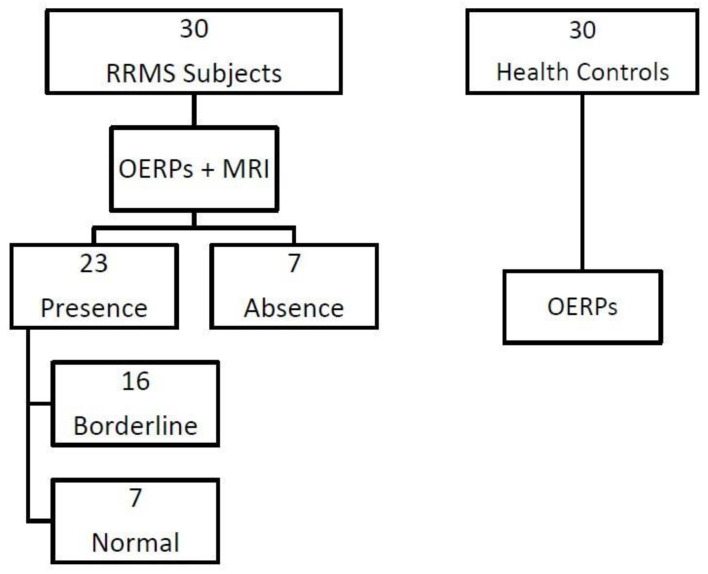
Study protocol and RRMS patients’ selection. **Legend:** OERPs = Olfactory Event Related Potentials; RRMS = relapsing-remitting multiple sclerosis; MRI = magnetic resonance imaging.

All subjects underwent an OERP examination according to the detailed protocol reported by Caminiti and coll. [4]. Table 2 shows mean OERP values computed on Fz, Cz, and Pz for each MS patient and normal control. All control subjects had normal amplitude values [12]. Seven of 30 RRMS patients showed a marked olfactory dysfunction, as detected by OERP absence, while 23 patients showed OERP responses. Of these latter patients, 16 had a strong reduction in N1-P2 component amplitude, but normal latency (borderline OERPs), compared with control subjects, compatible with a mild olfactory impairment, and the remaining 7 patients had normal latency and amplitude of N1 and P2 components (normal OERPs) [4]. Based on the OERP results, we divided the RRMS patients into 3 groups: a group with absent OERPs (AO group), a group with normal OERPs (NO group), and a group with borderline OERPs (BO group). These RRMS patients underwent MRI examination with gadolinium enhancement within a few days from OERP exam (Figure 1). An experienced neuroradiologist, unaware of the patient’s performance on OERP examination, performed MRI analysis. The study was conducted in accordance with the Declaration of Helsinki and was approved by the Ethic Committee of IRCCS Centro Neurolesi Bonino Pulejo (protocol code 06/2013); all subjects gave written informed consent.

### 2.3. Magnetic Resonance Imaging (MRI)

The patients underwent MRI with gadolinium enhancement using a scanner operating at 3.0 T (Achieva, Philips Healthcare, Best, the Netherlands) and a 32-channel SENSE head coil. For each subject, T1- [TR = 8 ms, TE = 4 ms, slice thickness/gap = 1/0 mm, number of slices = 173, field of view 240 mm] and T2-weighted [TR = 3.0 s, TE = 80 ms, slice thickness/gap = 3.0/0.3 mm, number of slices = 30, field of view 230 mm] sequences were acquired. The counting of the brain lesions was performed manually by an expert neuroradiologist.

### 2.4. Statistical Analysis

Statistical analysis was performed using an open source R3.0 software package. The analysis was conducted with descriptive statistics of demographic and clinical characteristics, followed by the mean and standard deviation of groups. Distribution of data was evaluated using the Shapiro–Wilk normality test. The χ^2^ test was used for comparison of the number of lesions in each area between MS groups with and without OERPs. Correlations between clinical variables (age, DD EDSS score, and OERP parameters) and number of lesions in each area were computed by Spearman’s coefficient. Finally, we performed a multiple regression analysis, which revealed the influence of OERPs parameters on the clinical variables (age, DD, and EDSS score) and the number of lesions (for total brain and its areas). Thus, we used OERP parameters as dependent variables, and clinical variables as predictors. We applied a backward elimination stepwise procedure for the choice of the best predictive variables according to the Akaike information criterion (AIC). If the number of predictors exceeded the sample size, we used a Fitting a Penalized Regression (Lasso) Model to automatically select the most relevant predictors, reducing the risk of overfitting and enhancing the reliability of estimates under limited sampling conditions. A 95% of confidence level was set with a 5% alpha error. Statistical significance was set at *p* < 0.05.

## 3. Results

### 3.1. Sample Analysis

Table 3 summarizes a more detailed description of the demographic, clinical, and imaging characteristics of RRMS patients divided into three groups. The χ^2^ test showed no significant association between the AO group and OERP presence group (BO + NO) and the number of lesions in each area, the frontal (χ^2^ = 0.07, *p* = 0.78), parietal (χ^2^ = 1.13, *p* = 0.29), temporal (χ^2^ = 1.69, *p* = 0.19), and occipital (χ^2^ = 0, *p* = 1) lobes. A significant association between the NO and AO groups and the number of lesions in the frontal area (χ^2^ = 16.4, *p* < 0.001), but not in the parietal (χ^2^ = 0.16, *p* = 0.69), temporal (χ^2^ = 2.76, *p* = 0.96), and occipital (χ^2^ = 0.18, *p* = 0.67) areas, was found. No significant association between the BO and AO groups and the number of lesions in each area, the frontal (χ^2^ = 0.02, *p* = 0.88), parietal (χ^2^ = 2.10, *p* = 0.15), temporal (χ^2^ = 0.82, *p* = 0.36), and occipital (χ^2^ = 0.14, *p* = 0.71) lobes, was found.

### 3.2. Spearman Correlation in BO Group

In the BO group, we observed a significant positive correlation between the number of lesions in the temporal area (r = 0.68; *p* = 0.03) and OERP parameters. Moreover, a significant trend between the number of lesions in the temporal area and N1 latency on Pz (r = 0.57; *p* = 0.08) and P2 amplitude on Fz (r = 0.54; *p* = 0.09) was found. No significant correlation between clinical characteristics and OERP parameters was found.

### 3.3. Spearman Correlation in NO Group

In the NO group, we observed a significant positive correlation between the number of lesions in the temporal area and OERP parameters. In particular, the number of lesions in the temporal area correlated with N1 latency on Fz (r = 0.87; *p* = 0.01), on Cz (r = 0.87; *p* = 0.01), and on Pz (r = 0.68; *p* = 0.04), and with P2 latency on Fz (r = 0.87; *p* = 0.01), Cz (r = 0.8; *p* = 0.03), and Pz (r = 0.87; *p* = 0.01). No significant correlation between clinical characteristics and OERP parameters was found.

### 3.4. Multiple Regression Analysis in BO Group

In Table 4, the results show that the clinical condition of the BO group had a significant impact on OERP parameters. The age and DD of the patients influenced the performance of P2 latency on Fz, whereas the total number of lesions and the number of lesions on the parietal area were significant predictors for P2 latency on Fz and P2 amplitude on Fz.

### 3.5. Multiple Regression Analysis in NO Group

In the NO group (Table 5), the number of lesions in the frontal area was a predictor for P2 amplitude on Fz and on Pz. Moreover, age, EDSS score, the number of lesions in parietal area, and the number of total lesions were predictors for P2 amplitude on Fz.

## 4. Discussion

This study investigated the association between the parameters of OERP components and the lesion load in brain regions involved in olfaction among RRMS patients. Our findings highlighted a significant association between the NO and AO groups and the number of lesions in the frontal area, underscoring the impact of frontal lobe lesions on olfactory processing in MS patients. This is consistent with previous studies that have linked frontal brain regions with olfactory function, suggesting that the lesions in these areas could disrupt olfactory pathways [7,9,13]. Interestingly, no significant associations in other brain regions (parietal, temporal, and occipital) were found, indicating a specific localization of olfactory processing disruptions in MS to the frontal area. This finding contrasts with the broader neural network typically associated with olfaction, which includes not only the frontal but also temporal region [7,9,13]. This discrepancy could be attributed to different methods to assess the olfaction. Indeed, Doty used UPSIT to assess the ability of 26 confirmed MS patients to identify odors, showing a clear connection between UPSIT scores and the number of plaques in the areas of the brain responsible for smelling [7]. This association was reported in a longitudinal study, linking the reduction and intensification of plaque quantity with UPSIT scores [13]. Zorzon used the Cross-Cultural Smell Identification Test to test the olfactory function of 40 patients with MS, finding a robust correlation between inferior-frontal and temporal lesion load and smell loss [9]. The literature data reported that more plaques reflect a worsening of olfactory performance [7,8,9]. Conversely, our results (Table 1) showed a higher lesion number in MS patients with OERPs than in patients without OERPs both in the global brain and its single areas. However, each SM patient of the AO group (seven patients) had a higher percentage of lesions (14.28%) compared to each of the 23 SM patients with OERPs (4.35%). In the NO group, a significant positive correlation was observed between the number of lesions in the temporal area and OERP parameters, particularly related to N1 and P2 latency. In the BO group, we observed a significant positive trend between the number of lesions in the temporal area and N1 latency and P2 amplitude. These results support Holinski’s study, which showed that there is a correlation between P2 latency and the volume, P2 latency, and quantity of MS lesion in the olfactory brain, including the piriform and enthorinal cortex, the front agranular region of the insular lobe up to the anterior commisure, and the orbital frontal cortex [10]. It could be that compensatory neuroplastic mechanisms are at play in patients presenting with OERP despite the presence of brain lesions. Neuroplasticity, that is, the brain’s ability to reorganize itself by forming new neural connections in response to injury, becomes crucial in maintaining sensory functions like olfaction in MS. One key compensatory mechanism involves the recruitment of additional brain regions not primarily associated with olfactory processing [14]. For instance, while the frontal lobe is central to olfactory function, damage in this area could induce the brain to engage other regions, such as the temporal and parietal lobes, to support olfactory processing. This recruitment likely occurs through the strengthening of existing connections or the creation of new pathways, allowing these regions to partially take over the role of the damaged frontal lobe. Moreover, increased neural activity in regions that remain intact might occur as a way to compensate for the loss of function in the damaged areas. In patients where OERPs are still present, the temporal lobe, for example, might exhibit heightened activity or faster processing times to help maintain the detection and interpretation of olfactory stimuli. This can be observed in the correlation between lesion load in the temporal area and specific OERP measures, suggesting that these areas are actively compensating for impaired olfactory processing. Synaptic plasticity also plays a role in these compensatory processes. The brain may enhance the efficiency of synaptic transmission in undamaged pathways, making them more responsive to olfactory stimuli. These hypotheses seem to be confirmed by OuYang [15], who, in a olfactory event-related fMRI study, reported a decreased activation of the olfactory-related brain networks in MS compared to control subjects, but also a varied activation of different brain areas that could indicate either heightened activity or a compensatory sense of smell process, akin to Parkinson’s disease [16] and Alzheimer’s disease [17]. Nevertheless, this aspect warrants additional examination. Finally, our results indicate that the clinical condition of the patients significantly impacts OERP parameters, with factors such as age and DD influencing the P2 latency. This suggests that beyond lesion load, patient-specific factors also play a crucial role in olfactory dysfunction. The predictive value of the number of lesions in the frontal and parietal areas for P2 amplitude also highlights the potential for OERP measures to serve as biomarkers for disease progression in MS. The cross-sectional design limits our ability to draw causal inferences between lesion load and olfactory dysfunction. Longitudinal studies would be beneficial to elucidate the progression of olfactory dysfunction in relation to lesion accumulation and disease progression in MS. Additionally, incorporating a broader range of olfactory tests, biochemical immune markers, and neuroimaging modalities could provide a more comprehensive understanding of the neural basis of olfactory dysfunction in MS. Given that the sample size is small, the findings should still be interpreted carefully and with caution. To reach final conclusions, the study must be expanded to include a bigger group of people.

## 5. Conclusions

Our findings contribute to the growing body of evidence on the neural basis of olfactory dysfunction in MS, highlighting the role of frontal brain lesions and the potential of OERP measures as sensitive markers of olfactory processing disruptions. These insights into the olfactory dysfunction in MS not only improve our understanding of the disease’s neurological impact but also open avenues for developing targeted interventions to mitigate sensory deficits in affected patients.

## Figures and Tables

**Table 1:** Description of demographic and clinical characteristics of patients and controls.

	RRMS Patients			Normal Controls		
	Females	Males	All	Females	Males	All
**Participants**	19 (63.33%)	11 (36.67%)	30 (100%)	18 (60%)	12 (40%)	30 (100%)
Age (mean ± SD)	35.21 ± 8.20	37.45 ± 4.04	36.03 ± 6.96	33.55 ± 9.39	39.25 ± 6.62	35.83 ± 8.74
EDSS (mean ± SD)	2.13 ± 1.21	2.00 ± 0.84	2.08 ± 1.07			
DD (mean ± SD)	5.31 ± 3.04	6.82 ± 3.63	5.87 ± 3.29			
**DMT**						
None	5 (16.67%)	4 (13.33%	9 (30%)			
Copaxone	1 (3.33%)	2 (6.67%)	3 (10%)			
Avonex	3 (10%)	1 (3.33%)	4 (13.33%)			
Rebif 22	2 (6.67%)	0 (0%)	2 (6.67%)			
Rebif 44	2 (6.67%	2(6.67%)	4 (13.34%)			
Gylenia	1 (3.33%)	0 (0%)	1 (3.33%)			
Tysabri	3 (10%)	1 (3.33%)	4 (13.33%)			
Extavia	2 (6.67%)	1 (3.33%)	3 (10%)			

**Legend:** DD = disease duration; DMT = disease modifying therapy; RRMS = relapsing-remitting multiple sclerosis; SD = standard deviation; EDSS = Expanded Disability Status Scale.

**Table 2:** Mean OERP values computed on Fz, Cz, and Pz for each MS patient and normal control.

	RRMS Patients			Normal Controls	
N1 Latency	P2 Latency	P2 Amplitude	N1 Latency	P2 Latency	P2 Amplitude
(Mean of Fz.Cz.Pz)	(Mean of Fz.Cz.Pz)	(Mean of Fz.Cz.Pz)	(Mean of Fz.Cz.Pz)	(Mean of Fz.Cz.Pz)	(Mean of Fz.Cz.Pz)
617.67	719.33	1.98	608.67	705.33	8.07
655.67	716.00	3.20	622.67	720.67	6.63
603.33	699.00	4.65	631.33	672.00	5.33
626.00	684.67	3.83	619.00	694.67	4.80
651.67	723.33	8.37	665.67	746.33	4.87
644.00	734.33	3.00	645.67	718.67	4.93
626.67	725.00	2.17	646.33	690.33	5.27
629.00	711.00	4.53	647.67	744.67	6.90
628.00	708.67	3.57	663.00	751.33	6.33
622.50	710.00	2.15	641.67	709.33	4.43
642.33	720.00	3.63	644.33	750.00	6.27
626.67	740.67	2.77	667.33	733.33	6.67
646.67	726.33	4.00	655.00	740.67	7.07
641.00	737.00	2.00	620.00	723.33	7.40
627.33	740.00	3.03	644.33	728.00	4.83
685.33	727.33	2.37	664.67	723.00	6.70
670.33	729.33	2.50	637.67	700.00	6.53
613.00	734.67	3.57	667.33	762.67	3.67
641.67	730.33	5.17	630.67	698.33	6.27
621.33	720.67	7.30	636.67	691.67	5.90
643.33	745.00	2.97	627.00	700.00	8.60
625.33	731.67	3.10	618.33	705.00	4.33
656.33	752.67	3.33	610.00	731.67	4.33
-	-	-	613.33	690.00	4.83
-	-	-	604.67	730.00	5.90
-	-	-	616.67	730.00	5.27
-	-	-	606.67	713.33	9.33
-	-	-	615.00	721.67	4.43
-	-	-	653.33	738.67	6.07
-	-	-	630.00	728.33	6.33

Values are in ms. Amplitude values are in mV.

**Table 3:** Demographic and clinical characteristics of 3 MS patient groups, frequencies (%).

	Present OERPs Absent OERPs (AO)		Present OERPs	
			Borderline OERPs (BO)	Normal OERPs (NO)
N. Subjects	23 (77%)	7 (23%)	16 (69%)	7 (31%)
Gender				
Male	7 (30.4%)	4 (57.1%)	6 (37.5%)	1 (14.2%)
Female	16 (69.6%)	3 (42.9%)	10 (62.5%)	6 (85.8%)
Age (mean ± SD)	43.4 ± 8.19	56.14 ± 8.93	44.43 ± 8.60	42.0 ± 7.71
DD (mean ± SD)	5.22 ± 3.48	8.0 ± 1.0	5.5 ± 3.84	5.0 ± 2.99
EDSS (mean ± SD)	1.96 ± 1.12	2.50 ± 0.87	1.75 ± 1.05	2.28 ± 1.20
Number Lesions	104 (74.3)	36 (25.7)	62 (59.6)	47 (45.2)
Frontal	39 (37.5)	12 (33.3)	21 (33.9)	17 (36.2)
Parietal	29 (27.9)	10 (27.8)	14 (22.6)	14 (29.8)
Temporal	28 (26.9)	11 (30.6)	14 (22.6)	10 (21.3)
Occipital	8 (7.7)	3 (8.3)	7 (11.3)	6 (12.7)

**Legend:** N. Subjects = number of subjects; DD = disease duration; SD = standard deviation; EDSS = Expanded Disability Status Scale; OERPs = Ongoing Event-Related Potentials; AO = absent OERP; BO = borderline OERP; NO = normal OERP.

**Table 4:** Backward linear regression in BO group: significant predictors on OERP parameters.

Dependent Variables	Predictors	*β*	Std *β*	df	95%CI		*p*-Value	Adjusted R2
					Lower	Higher		
P2 latency Fz	Age	−4.39	−1.51	7	−7.96	−0.82	0.01	0.78
	DD	5.4	0.79	7	3.53	7.27	0.06	
	Total Lesions	53.16	1.66	7	49.23	57.08	0.03	
	Parietal Lobe	−91.83	−0.62	7	−93.30	−90.36	0.01	
P2 amplitude Fz	Total Lesions	1.49	1.31	7	−1.61	4.59	0.5	0.77
	Parietal Lobe	−2.15	0.02	7	−2.20	−2.10	0.05	

**Legend:** *β* = regression coefficient; Std *β* = standardized regression coefficient. DD = disease duration.

**Table 5:** Backward linear regression in NO group: significant predictors on OERP parameters.

Dependent Variables	Predictors	*β*	Std *β*	df	95%CI		*p*-Value		Adjusted R2
					Lower	Higher			
P2 amplitude Fz	Age	−0.35	−1.83	1	−4.56	3.87	0.04	0.99	
	EDSS	−4.23	−2.51	1	−23.60	22.90	0.06		
	Frontal Lobe	−6.49	−1.47	1	−25.17	12.18	0.04		
	Total Lesions	−2.54	−2.37	1	−32.65	27.57	0.08		
P2 amplitude Fz	Total Lesions	−10.9	−1.46	1	−29.45	7.65	0.09	0.94	

**Legend:** *β* = regression coefficient; Std *β* = standardized regression coefficient. EDSS = Expanded Disability Status Scale.

## Data Availability

The data presented in this study are available on request from the corresponding author.

## Abbreviations

The following abbreviations are used in this manuscript:

AO	Absent OERP
BO	Borderline OERP
DD	Disease duration
EDSS	Expanded Disability Status Scale
MRI	Magnetic resonance imaging
MS	Multiple sclerosis
NO	Normal OERP
OD	Olfactory dysfunction
OERPs	Olfactory Event-Related Potentials
